# Surrogate Perspectives on the Communication and Support Processes That Enable Them as Active Decision-Makers Across Chronic Critical Illness

**DOI:** 10.1016/j.chstcc.2025.100220

**Published:** 2026-02-11

**Authors:** Amanda C. Moale, Douglas B. White, Bryan J. McVerry, Robert Grier, Chareeni E. Kurukulasuriya, Mikhaila N. Layshock, Svea Cheng, Robert M. Arnold, Maya I. Ragavan, Renee D. Boss, Judy C. Chang

**Affiliations:** Division of Pulmonary, Allergy, Critical Care and Sleep Medicine, Department of Medicine (A. C. M. and B. J. McV.), University of Pittsburgh School of Medicine; the Department of Critical Care Medicine (D. B. W. and B. J. McV.), University of Pittsburgh School of Medicine; Family partner (R. G.), Pittsburgh, PA; the Department of Otolaryngology-Head and Neck Surgery (C. E. K.), New York University Grossman School of Medicine, New York City, NY; the Department of Medicine (M. N. L. and S. C.), University of Pittsburgh School of Medicine, University of Pittsburgh, Pittsburgh, PA; the Department of Geriatrics and Palliative Medicine (R. M. A.), Icahn School of Medicine at Mount Sinai, New York City, NY; the Department of Pediatrics (M. I. R.), University of Pittsburgh School of Medicine, Pittsburgh, PA; the Department of Pediatrics (R. D. B.), Johns Hopkins University School of Medicine, Baltimore, MD; and the Departments of Obstetrics, Gynecology and Reproductive Sciences, Internal Medicine, and the Clinical and Translational Science Institute (J. C. C.), UPMC Magee-Womens Hospital, Pittsburgh, PA.

**Keywords:** chronic critical illness, family caregiving, persistent respiratory failure, prolonged mechanical ventilation, surrogate decision-makers, tracheostomy, decision making, shared

## Abstract

**BACKGROUND:**

Chronic critical illness (CCI) is marked by persistent respiratory failure, with patients commonly receiving tracheostomies for ongoing ventilation across ICU and post-ICU settings. Well-documented communication and support breakdowns across CCI worsen family distress and may perpetuate care misaligned with patient values and goals. Yet, effective interventions supporting surrogate decision-makers throughout CCI are lacking.

**RESEARCH QUESTION:**

What are the communication and support processes that families view as essential to enabling them as active surrogate decision-makers across the patient’s trajectory to better inform future intervention development?

**STUDY DESIGN AND METHODS:**

We conducted semistructured interviews with surrogate decision-makers of patients who received a tracheostomy for persistent respiratory failure after an acute illness at 2 time points (first, 2 weeks to 6 months after tracheostomy; second, weeks to months later). We used deductive and inductive coding, followed by thematic analysis of interview transcripts.

**RESULTS:**

We interviewed 23 surrogate decision-makers of 19 medical and surgical patients with moderate-high illness severity (mean Acute Physiology and Chronic Health Evaluation II score, 21.5 ± 5.3). Five themes were generated from family perspectives, representing the essential communication and support processes to enable families as active decision-makers across time and settings in CCI. They included being (1) engaged proactively during transitions; (2) connected to tailored practical resources that enable their presence, engagement, and space for self-care across the CCI trajectory; (3) actively engaged in ongoing and iterative anticipatory guidance and reassessment; (4) recognized as the expert and advocate for the patient; and (5) treated with compassion and respect.

**INTERPRETATION:**

Our findings suggest that future interventions should deliver decisional support in CCI through longitudinal, relationship-centered strategies integrating iterative care planning, compassionate and inclusive family engagement, and tailored practical resources as core intervention components.

Families play a central role in the care of patients with chronic critical illness (CCI), a syndrome marked by persistent respiratory failure and prolonged dependence on mechanical ventilation after an acute illness. ^1^ Patients often receive tracheostomies to facilitate ongoing mechanical ventilation and care across ICU and post-ICU settings, with clinicians often recognizing tracheostomy placement as the transition from acute illness to CCI. Therefore, in this article, we specifically refer to the subtype of patients with CCI who received a tracheostomy for persistent respiratory failure after an acute illness. These patients are often incapacitated for extended durations and rely on others to make medical decisions on their behalf. ^1,2^ However, communication and support breakdowns are common across time and settings in CCI, contributing to family distress and care that may conflict with the patient’s values and goals. ^3–8^

Few randomized trials have specifically tested family communication and support interventions in CCI. One evaluated the effect of a decision aid for prolonged mechanical ventilation and another tested structured palliative care-led family meetings. ^9,10^ Despite being well designed, neither improved patient or family outcomes. Nevertheless, both interventions were limited to the ICU setting and therefore could not address the communication and support problems beyond the ICU. Moreover, Black families and those from rural communities face disparities in access, quality of care, and shared decision-making, underscoring the importance of inclusive family engagement.^11,12^

Our objective was to specify the communication and support processes that families consider most important for effectively serving as surrogate decision-makers across the patient’s CCI trajectory, to better inform the design of future family communication and support interventions in CCI.

## Study Design and Methods

### Study Design, Participant Selection, and Setting

Data presented in this article come from a longitudinal qualitative study using a qualitative description approach to explore the perspectives of surrogate decision-makers across time and care settings in CCI. ^5^ The methods have been previously detailed. ^5^ Our research team included a family representative (R. G.) with lived experience as a caregiver and surrogate decision-maker for a relative with CCI and dependence on mechanical ventilation. He was actively involved in data analysis and interpretation.

Eligible participants were actively involved in decision-making for adult patients who received a tracheostomy in an ICU for persistent respiratory failure following an acute illness. We excluded family members of patients who received an emergency tracheostomy or were lung transplant recipients. Inclusion of multiple family members of the same patient was permitted if each met eligibility criteria, recognizing the collaborative nature of decision-making.

We purposively sampled to ensure that at least 20% of participants were from rural communities and 20% identified as Black or multiracial, as these groups experience disparities and health care access challenges. Black families also face racial inequities in shared decision-making. ^11^

### Institutional Review Board Information

For recruitment, we obtained permission from the patient’s physician before approaching families of ICU patients at 3 hospitals in western Pennsylvania, either in person or by telephone, to assess eligibility, explain the study, and invite participation. This study was approved by the University of Pittsburgh institutional review board (STUDY22060144). All participants provided informed consent and were offered compensation. We have reported our study in accordance with the Consolidated Criteria for Reporting Qualitative Research (COREQ). ^13^

### Data Collection

Families engaged in 2 1-hour semistructured interviews conducted by telephone or in person. The interview guides (e-Appendixes 1, 2) covered three main topics: (1) experiences around decision-making for tracheostomy; (2) broader experiences with CCI up to the time of the interview; and (3) the information, resources, and supports that families viewed as most important over time and across care transitions. The second interview guide revisited these 3 topics but focused on the perspectives of each family and their reflections at a later point in the patient’s care journey. Data from the first 2 topics elucidated critical problems in communication and support across the CCI continuum and highlighted that tracheostomy was only 1 of many decisions families faced.^5^ This article presents findings from the third topic—information, resources, and supports that families identified as essential to enabling them as surrogate decision-makers. We pilot tested the interview guides with content and methodologic experts, revising them on the basis of feedback to ensure clarity and relevance.

Interviews were conducted between January 2023 and September 2024. The first interview was scheduled 2 weeks to 6 months after tracheostomy placement, with the second interview scheduled weeks to months later. All interviews were conducted by a trained interviewer and pulmonary and critical care physician (A. C. M.), audio-recorded, and transcribed verbatim with identifying information removed.

Families also completed a survey that included demographic questions, along with several measures with validity evidence (e-Appendix 3),^14–17^ and we extracted patient clinical and contextual variables from the electronic medical record. We concluded recruitment after reaching thematic saturation, defined as the point at which no new codes or themes were generated,^18,19^ and at least 20% of families were from rural communities and identified as Black or multiracial.

### Data Analysis

Interview transcripts were analyzed using the Crabtree and Miller^19^ template approach to qualitative text analysis, which is suited for combing predefined and inductive codes. Two coders, A. C. M. and C. E. K., first familiarized themselves with the data and then developed a preliminary codebook template that included predefined codes based on the interview guides and the 3 stages of shared decision-making—information exchange, deliberation, and decision-making.^20^ However, most codes for the data in this article were developed inductively from the interview content itself.^21^

As interviews were completed and transcripts were generated, A. C. M. and C. E. K. independently coded the first 16 transcripts (approximately 41%), applying the preliminary codebook and inductively creating new codes to capture emerging topics. Coders met regularly to compare coding, discuss interpretations, achieve consensus, and iteratively revise the codebook. To ensure consistent application of codes, each code was accompanied by a definition and rule for application. New or revised codes were also applied to earlier transcripts. Any major discrepancies were to be adjudicated by a third investigator; however, none arose. We continued dual coding until new codes were infrequently added, and both coders were consistently applying existing codes. At that point, A. C. M. coded the remaining transcripts, continuing to make minor revisions and additions as necessary. The codebook was finalized when no further edits or additions were required.

As transcripts were coded, A. C. M. and R. G. (family representative) organized and interpreted the data by identifying patterns and categories, and generating themes, which were iteratively refined as data collection progressed and with the full research team. Results were returned to research participants via an infographic, and feedback was elicited through a brief survey. On the basis of feedback, themes were revised to appropriately represent participant perspectives. We used NVivo 12 software (version 12.7; Lumivero) for qualitative data management. ^22^

## Results

### Participants

We enrolled 23 surrogate decision-makers of 19 patients, with 22 completing the initial interview and 17 completing the second interview. As permitted by our inclusion criteria, more than 1 family member was included for 2 patients. To accommodate participant preferences, 2 family members also participated jointly in 1 interview.

The median family member age was 51 years (interquartile range [IQR], 43–58), with most being adult children (47.8%) or spouses/partners (39.1%), 25% identifying as Black or multiracial, and 28.6% residing in rural areas. The median patient age was 61 years (IQR, 55–72) and included a mix of patients initially admitted to a medical unit (47.4%) or surgical unit (52.6%). The median days since tracheostomy was 26 days (IQR, 15–38) at interview 1 and 114 days (IQR, 85–173) at interview 2, with a median of 71 days (IQR, 53–152) between interviews. Family characteristics are shown in Table 1, with corresponding patient characteristics in Table 2.

### Themes

**Figure 1 –:**
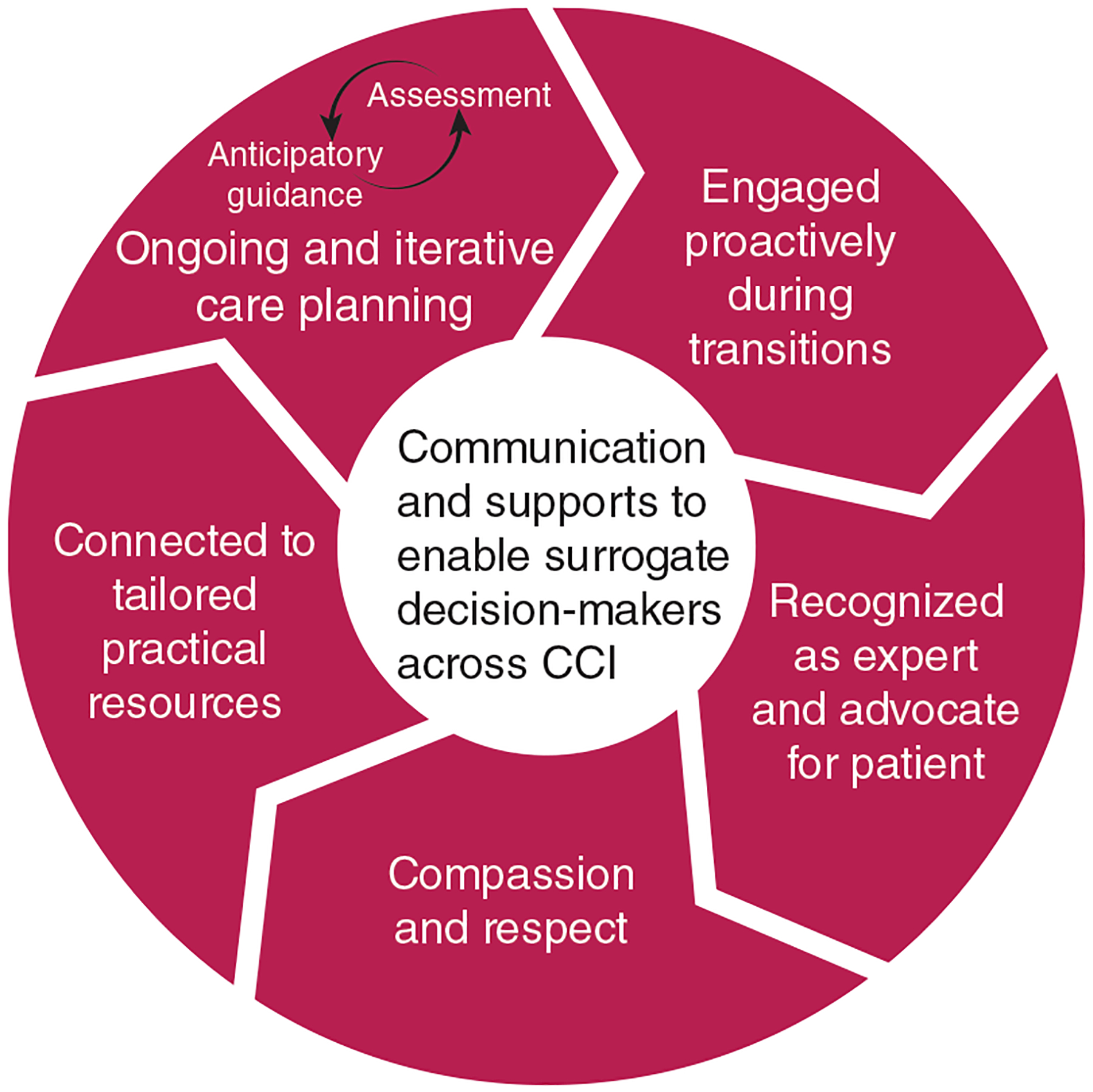
Core communication and support processes enabling families to serve as effective surrogate decision-makers across chronic critical illness (CCI).

Five themes were generated from family perspectives, representing the essential communication and supports processes to enable families as active decision-makers across time and settings in CCI (Fig 1). Families emphasized the importance of being (1) engaged proactively during transitions; (2) connected to tailored practical resources that enable their presence, engagement, and space for self-care across the CCI trajectory; (3) actively engaged in ongoing and iterative anticipatory guidance and reassessment; (4) recognized as the expert and advocate for the patient; and (5) treated with compassion and respect.

#### Theme 1: Engaged proactively during transitions:

Although families identified care transitions as times when active engagement was critical, they were also the most frequently cited periods when communication and care coordination unraveled. Consequently, during transitions, families often felt overlooked, like the new care team did not “know” the patient, and that the patient’s care plan was depersonalized. As 1 family member stated, “*Each time you move from place to place*…*we don’t know her doctor and the doctor doesn’t know her*…*yet [they] are making adjustments and decisions*…*that they think might fit with a woman of her age and size*…” (P1).

Most families recognized that care transitions come with new clinicians and care environments, but many reported that their uncertainty and anxiety worsened when they were not engaged early or included in care plan coordination with the new care team. Although clinicians typically receive sign-out from the previous team, families emphasized the importance of being engaged early by the care team so they could confirm what information about the patient had been communicated between teams, help fill in any gaps about the patient, and ensure alignment on the care plan moving forward. One participant shared, “*After he was transferred, everything changed, and people weren’t talking*” (P19). Another stated, “*You don’t have continuity and*…*we’ve had multiple experiences where things fell through the cracks*” (P3). Most families did not recall a longitudinal care plan being instituted in the ICU; for the few that did, they expressed frustration when the care plan was not followed or was changed without their active involvement. “*It seemed like everything was planned*…*but then she got moved to [a long-term acute care hospital] where that [plan] didn’t happen*” (P17).

Some families suggested having a dedicated point of contact throughout the patient’s CCI journey, citing palliative care and the post-cardiac arrest teams as examples, both of which follow patients and families longitudinally in our hospital system—from the ICUs and inpatient floors to affiliated long-term acute care hospitals, and, if applicable, home. “*I wish that everyone had a constant person*” (P1). “*It would be helpful to have someone who already knows the situation*…*so that you’re not sharing the same thing with a stranger all the time and they might have more empathy towards you since they already known you*” (P10).

#### Theme 2: Connected to tailored practical resources that enable their presence, engagement, and space for self-care across the CCI trajectory:

Most families said their top priority was being present and engaged in their family member’s care. Two participants emphasized this point, stating: “*What I need is to take care of her*” (P5), and “*When I’m worrying about my mother, I can’t take a moment for myself*” (P18). However, families encountered a range of structural and logistical barriers that made it difficult to be present and engaged. As examples, multiple families described how financial burdens, inadequate leave policies, and restricted visiting hours caused distress and prevented them from being physically present with their family member. One stated, “*[The biggest thing] is that I can’t get out there as much as I want*… *[because] everything is so expensive, and they don’t have a place for [family] to stay near the [facility]*” (P22). Another shared, “…*I have to go back to work because I’ve spent all my FMLA*….*There is no family that will be [there]*…*that is probably the biggest worry I have right now*” (P20).

Families described how practical resources tailored to their needs—lodging, parking vouchers, flexible visiting hours, protected leave, and financial resources—not only enabled them to be present and focused on their family’s care but also created space for their own self-care. Although many participants relied on friends, family, and their local communities to help manage competing responsibilities, not all had strong social networks. Even among those who did, many found that support often waned over time, making practical resources especially important. Families also emphasized the importance of practical resources after care transitions, when they were often unaware of what resources were available in the new setting. As 1 shared after a care transition, “*I didn’t realize that we could get [lodging] at a discount*…*[or] vouchers for food*” (P14).

#### Theme 3: Actively engaged in ongoing and iterative anticipatory guidance and reassessment:

Families emphasized that decision-making in CCI should be part of continuous care planning with repeated cycles of anticipatory guidance and reassessment across time and settings, rather than a series of seemingly isolated decisions focused on immediate concerns.

Before tracheostomy, one family member reflected on the importance of understanding the range of long-term implications and considering whether the patient’s goals could be met on their most likely path afterward: “*Show the spectrum of life after trach, some people who are doing rehab*…*.and others who are never getting rid of that trach*…*and [then explain] where the [patient] will most likely be on the spectrum*” (P11). Several others emphasized the importance of long-term anticipatory guidance. One family member said, “*We [also need] to understand*…*the things that could happen next*” (P17). Likewise, “*I understand being focused on the moment*…*but what about the steps afterwards?*” (P20). Even in times of uncertainty, families at least wanted best- and worst-case long-term scenarios: “…*I was grasping for any kind of ballpark*…*worst*…*best*” (P1).

Beyond wanting a clearer sense of what to expect before tracheostomy, families emphasized that anticipatory guidance and reassessment of the patient’s clinical status should be ongoing. Specifically, after tracheostomy, they wanted to understand the key milestones for recovery—including indicators of improvement, stagnation, or deterioration—before their next touchpoint with the clinical team. Understanding these milestones helped them gauge the patient’s progress and emotionally prepare for future discussions and decisions. Given the unfamiliarity of most families with long-term acute care hospitals, this transition was often a time when families sought to revisit anticipatory guidance and milestones, followed by reassessment of progress. As 1 family member shared after transfer to a long-term acute care hospital, “…*What would it mean if she can’t get off the [ventilator]*…*I need to know what happens next so we can start to prepare emotionally*” (P3). Similarly, another shared, “*[What if] they can’t take that next step?*” (P11).

#### Theme 4: Recognized as the expert and advocate for the patient:

Families wanted to be recognized for their unique and essential role in the care team, as they were often the only ones who truly knew and cared about the patient as a person. They emphasized that their knowledge of the patient and their values was critical to the team providing the right care. “*I can’t expect medical professionals who barely know him at all to*… *[tell me] what a fulfilling life for him would be*” (P10). Yet, several felt unrecognized or unwelcomed by the clinical team, leaving them feeling invisible or undervalued. As 1 shared, “…*It was as if the patient was there by themselves*” (P20). They often had to repeatedly assert their presence, “…*I had to announce myself each time [in order to be recognized]*” (P20). One family member noted how even simple acknowledgment can shift the dynamic: “*It’s such a simple thing to do. When doctors or nurses come in: ‘Hi, how are you? How’s everything today? What’s your relationship?’ Bam, now you know they’re their caregiver*” (P21).

#### Theme 5: Treated with compassion and respect:

Families also emphasized the importance of the more general interpersonal interactions of clinicians with them and the patient. When families were asked what helped them the most or how they wished they were supported during challenging times and decisions, their first response was most often about how the care team treated them or the patient. Families who felt that the clinical team was compassionate, respectful, and empathetic toward both them and the patient described a stronger sense of trust in the team and overall comfort. As one stated, “*It wasn’t just a business*…*I would trust them with my health*” (P8). Similarly, “*Being patient, courteous, and understanding*…*goes a long way of making me feel at ease*” (P4). Another family shared how compassionate care made them feel that the team was genuinely invested in the patient’s well-being: “*There has been this personal touch from day one*…*I firmly believe that they think and talk about her even when they’re not here*” (P1).

## Discussion

Families identified the essential communication and support processes that enable them to stay engaged, advocate for the patient, and prepare for and navigate decisions throughout CCI. Families stressed that their ability to serve as surrogate decision-makers depends on being proactively engaged during transitions; supported with practical, tailored resources; engaged in ongoing and iterative care planning; recognized for their crucial role; and treated with respect and compassion. Our findings offer new, actionable guidance for how to deliver communication and support both in clinical practice and future CCI interventions across time and settings. Yet, current health care systems are not structured to support the continuity and relational engagement families described as essential intervention features, underscoring the need for scalable strategies to deliver decisional support throughout the patient’s illness journey.

We found that the family-identified communication and support processes align with the Street and colleagues ^6^ communication functions to health outcomes model, which is important because it outlines pathways through which these processes can influence health outcomes. Although the Street model includes key communication functions—information exchange, responding to emotions, managing uncertainty, fostering relationships, making decisions, and enabling self-management—our findings add specificity by illustrating how families want these functions to be delivered in the context of CCI. For instance, to support self-management, they highlighted the importance of tailored, practical resources. To foster relationships, families emphasized the need to be compassionately engaged and acknowledged for their essential role. They also described information exchange, decision-making, and managing emotions as together constituting ongoing and iterative care planning.

In addition to how families want communication and support delivered, they underscored that the timing and settings in which these are delivered are key. Although much of the CCI literature has focused on the decision to pursue tracheostomy, ^9,23–28^ our findings highlight that pretracheostomy decision-making represents only 1 moment within a broader, iterative, and longitudinal process of relationship-centered care planning. Likewise, the ICU is just 1 care location for many patients with CCI receiving prolonged mechanical ventilation. Paul and colleagues ^29^ found that these patients experience a median of 3 care place transitions within 6 months after ICU discharge. Therefore, interventions limited to the ICU cannot address the major communication and support challenges that arise during transitions or support ongoing care planning. Importantly, fragmented communication and collaboration across transitions contributes to poor outcomes, including goal-discordant care, prolonged hospital stays, and acute care readmissions. ^30–33^

Families also emphasized that access to practical resources was crucial for facilitating family engagement and attending to their own self-care. Our findings are corroborated by existing literature describing how practical resources foster a sense of safety, connectedness, self-efficacy, and hope, ultimately creating space for families to attend to their own needs.^34,35^ Although some structural barriers, such as limitations in protected leave, are difficult to modify, the lack of family awareness and connection to available resources is an addressable gap. There may be opportunities to leverage interdisciplinary team members, including social workers, case managers, and nurses, to help assess and address the practical needs of families, particularly after care transitions when care needs and available resources often change. Moreover, tailoring practical resources on the basis of the varying financial, geographic, and social circumstances of families also promotes more equitable care.

Last, the emphasis placed by families on being respected and recognized as clinical resources and partners aligns with Chochinov’s elements of intensive caring, which underscore that ongoing support grounded in nonabandonment and relational affirmation foster family presence and achievable goal setting. ^36^ Empathy has also been found to predict emotional resilience and positively affect decision-making quality.^37,38^

Nevertheless, ongoing, relational, and iterative care planning across CCI is not well supported by current health care structures and cannot be addressed through 1-time or location-based interventions. Although such strategies have not been implemented in adult CCI, other fields managing prolonged illnesses, including oncology and pediatrics, have approached decision-making as a longitudinal, collaborative process. ^39–41^ In oncology, for example, embedding a lay health worker into routine cancer care to help patients navigate care preferences over 6 months improved goals documentation and satisfaction, and reduced costs. ^40^ Likewise, greater involvement of primary care providers during hospitalization may provide the relational continuity families seek. ^42^ However, deploying onsite personnel across multiple care settings or involving primary care providers may not always be feasible. Alternatively, technology-enabled interventions offer a more scalable path forward and are already in use across numerous disciplines. ^43–47^ For instance, Trivedi et al ^43,44^ developed and pilot tested telephone- and web-based self-management programs for patients with heart failure and their caregivers, improving self-management, communication, relationship quality, and caregiver burden. An interactive web-based tool more specifically designed to help surrogates prepare for clinician-family meetings has also been found feasible and acceptable among surrogates of critically ill patients. ^48^ Thus, adapting similar technology-enabled interventions for CCI may offer a feasible, scalable solution to improving patient and family outcomes in CCI. We also urge that future interventions be adapted or developed with families and other stakeholders to ensure they meet the needs of families and are implementable across settings.

Our study has several strengths. The longitudinal study design allowed us to capture the evolving perspectives of families across the patient’s illness journey and care settings. To our knowledge, this is also the first study to specify how families want key communication and support processes to be delivered across time and settings in CCI. Our findings offer actionable insights that may inform both clinical practice and future intervention design.

Our study also has several limitations. Although we recruited participants from 3 different hospitals, families receiving care at other institutions or regions may have different experiences and perspectives. In addition, patient mortality at the time of the second interview was lower in our cohort as compared with other reported CCI cohorts, and families of patients who died may have different insights.^1,49,50^ It is possible that the 18.2% of families lost to follow-up were those of patients with worse outcomes, including death, which may have contributed to an artificially low observed mortality. Last, although our cohort included a balanced representation of male and female individuals, varied education levels, 25% identifying as Black or multiracial, and 28.6% residing in rural areas, racial diversity was overall low, and all patients spoke English, which limits transferability. Although we did not observe thematic divergence by patient or family characteristics, divergences may have arisen if thematic saturation were assessed within specific subgroups.

## Interpretation

Future interventions should consider implementing longitudinal strategies that prioritize iterative care planning and cultivate inclusive, compassionate engagement with families, enabling them as surrogate decision-makers across the patient’s CCI trajectory and aiming to move the needle on patient- and family-centered outcomes in CCI.

## Supplementary Material

1

2

3

**Additional information:** The e-Appendixes are available online under “Supplementary Data.”

## Figures and Tables

**TABLE 1]:** Surrogate Decision-Maker Characteristics (n = 23)

Characteristic	Value
Age, y	51 (43–58)
Self-identified gender	
Women	13 (56.5)
Men	10 (43.5)
Self-identified race^a^	
Black	4 (20)
White	15 (75)
Multiracial	1 (5)
Highest level of education^b^	
High school or less	8 (38.1)
Some college	7 (33.3)
4-year degree or more	6 (28.6)
Residential setting ^b,c^	
Urban	15 (71.4)
Rural	6 (28.6)
Relationship to patient	
Adult child	11 (47.8)
Spouse or partner	9 (39.1)
Sibling	3 (13.1)
Brief Health Literacy Screen^b ,d^	
Limited	1 (4.7)
Marginal	3 (14.3)
Adequate	17 (81.0)
Decision Regret Scale ^b,e^	10 (0–20)
Subjective Numeracy Scale^b ,f^	36 (33–43)
Wake Forest Physician Trust Scale ^b,g^	40 (35–48)
Religion or spirituality is important to my life, (% important)^b ,h^	16 (76.2)
Religion or spirituality influenced the decision for tracheostomy, (% agree)^b ,h^	4 (19.0)
Religion or spirituality helped me cope with my family member’s illness, (% agree)^b ,h^	17 (81.0)

Data are presented as No. (%) or median (IQR) unless otherwise indicated. IQR = interquartile range.

a Missing data for 3 family members.

b Missing data for 2 family members.

c Based on ZIP code.

d Brief Health Literacy Screen values range from a minimum of 4 to a maximum of 20, with 4–12 interpreted as limited, 13–26 as marginal, and 17–20 as adequate health literacy.

e Decision Regret Scale values ranging from 0 to 100, with 0 meaning no regret and 100 meaning high regret.

f Subjective Numeracy Scale values ranging from 8 to 48, with higher scores indicating higher subjective numeracy.

g Wake Forest Physician Trust Scale values range from 5 to 50, with higher scores indicating more trust.

h Questions on a 1–5 Likert scale, with categories 4 and 5 recoded as “important” or “agree.” *Adapted with permission of the American Thoracic Society*. *Copyright*© *2025 American Thoracic Society. All rights reserved. Moale et al*.^5^

**TABLE 2]:** Corresponding Patient Characteristics (n = 19)

Characteristic	Value	
Age, y	61 (55–72)	
Sex		
Male	10 (52.6)	
Female	9 (47.4)	
Race from electronic medical record		
Black	5 (26.3)	
White	14 (73.7)	
Etiology of prolonged respiratory failure		
Acute on chronic respiratory failure	8 (42.1)	
Pneumonia	4 (21.1)	
Cardiogenic pulmonary edema	3 (15.8)	
Encephalopathy		
Hypoxic ischemia	3 (15.8)	
Encephalitis	1 (5.3)	
Toxic-metabolic	1 (5.3)	
Leptomeningeal carcinomatosis	1 (5.3)	
Traumatic brain injury	1 (5.3)	
Pulmonary embolism	1 (5.3)	
Admitting ICU		
Surgical	10 (52.6)	
Medical	9 (47.4)	
APACHE II score, mean (SD)^a^	21.5 (5.3)	
Insurance		
Medicare	12 (63.2)	
Commercial	3 (15.8)	
Medicaid	2 (10.5)	
Veterans Administration health care	2 (10.5)	
Patient status at time of completed interviews	Interview 1 ^b^	Interview 2 ^c^
Days since tracheostomy, d	26 (15–38)	114 (85–173)
Patient location at time of interview		
Floor	7 (31.8)	3 (17.6)
ICU	10 (45.5)	2 (11.8)
Long-term acute care hospital	5 (22.7)	0 (0)
Skilled nursing facility	0 (0)	4 (23.5)
Inpatient rehabilitation	0 (0)	1 (5.9)
Home	0 (0)	5 (29.4)
Deceased	0 (0)	2 (11.8)
Alive^d^	19 (100)	17 (89.5)

Data are presented as No. (%) or median (IQR) unless otherwise indicated. APACHE = Acute Physiology and Chronic Health Evaluation; IQR = interquartile range.

a APACHE II score from first available data during ICU stay when tracheostomy was performed. Missing data for 2 family members.

b n = 22, as 2 patients had more than 1 more family member.

c n = 17, as 5 family members were lost to follow-up or declined second interview.

d Status of alive or deceased based on total number of 19 patients. *Adapted with permission of the American Thoracic Society. Copyright © 2025 American Thoracic Society. All rights reserved. Moale et al*.^5^

## Take-Home Points

**Research Question:** What are the communication and support processes that families consider most important for effectively serving as surrogate decision-makers across the patient’s chronic critical illness trajectory?

**Results:** Families identified 5 essential processes to enable them as active decision-makers across chronic critical illness: 1) proactive engagement during transitions; 2) connection to tailored practical resources that enable their presence, engagement, and space for self-care across the chronic critical illness (CCI) trajectory; 3) ongoing, iterative anticipatory guidance and reassessment; 4) recognition as the expert and advocate for the patient; and 5) compassion and respect.

**Interpretation:** Future interventions should deliver longitudinal, relationship-centered decisional support in CCI that integrates iterative care planning, compassionate and inclusive family engagement, and tailored practical resources as core intervention components.

## ABBREVIATIONS:

CCI: chronic critical illness
