# Supplementary material for: Surrogate Perspectives on the Communication and Support Processes That Enable Them as Active Decision-Makers Across Chronic Critical Illness
Source: CHEST Crit Care. Author manuscript; Available in PMC 2026 Apr 9. (PMC13060019; doi:10.1016/j.chstcc.2025.100220)
Supplement: 1 [file NIHMS2157069-supplement-1.docx]

**Understanding Perspectives of Surrogate Decision Makers of Patients who Received Tracheostomies for Prolonged Mechanical Ventilation**

**Question guide:**

1. Tell me a little about your family member as a person.
   1. Before their illness, what did they like to do?
   2. What is your relationship with them like?
   3. What, if anything, had they said would be most important to them if they were to become seriously ill?
   4. What were you told about why they were put on a ventilator? Why were they having trouble getting off?
2. A tracheostomy creates an opening through the front of the neck and allows people who cannot breathe on their own to be connected to a ventilator. Before deciding whether your family member should get a tracheostomy, what were your ideas about what this procedure was for?
3. What did the health care team tell you about tracheostomy leading up to the decision?
   1. What options did the team describe? (incl. what did they talk about if you decided not to do a trach?)
   2. What did they tell you about how life would look after tracheostomy? What else?
   3. Tell me about any information that was confusion from the health care team about getting a tracheostomy. Contradictory?
4. What questions did you have about your family member getting a tracheostomy and staying connected to the ventilator?
   1. How did you find the answers to those questions?
   2. Who else outside of the healthcare team did you talk to about this situation? Who else?
   3. Did you look at the internet? Tell me what you found.
5. How was the decision for tracheostomy made?
   1. What made this decision harder? Easier?
   2. What recommendations did the healthcare team give?
   3. Prior to making the decision about tracheostomy, what was your most important hope for your family member at the time? What concerns or worries did you have?
6. Tell me about how things have been since your family member received a tracheostomy?
   1. What have the biggest challenges been?
   2. How is your family member different now compared to how they were before they went to the ICU?
   3. Tell me about if life has been like you thought it would be after tracheostomy? What is different than you thought it would be?
7. What have you been doing to care for yourself?
8. If someone else was considering a tracheostomy for their family member, what would you tell them?
   1. How can we best prepare them for life after tracheostomy?
      1. What information do you wish you had known prior to tracheostomy? (i.e., risks, implications, prognosis, outcomes).
      2. What information, if any, would have been helpful even before your family member got sick?
      3. What resources, services and/or support do you wish you had when making that decision?
9. Reflecting back on the decision to go ahead with a tracheostomy, is there anything else you wish the health care team would have done differently? Anything you would have done differently?
10. What else should I know about this topic that I haven’t thought to ask?

**Conclusion:** We really appreciate all your thoughts and input. All your comments are private and confidential. They are important for making care better for people like you and your family member. Thank you again.
