# Supplementary material for: Surrogate Perspectives on the Communication and Support Processes That Enable Them as Active Decision-Makers Across Chronic Critical Illness
Source: CHEST Crit Care. Author manuscript; Available in PMC 2026 Apr 9. (PMC13060019; doi:10.1016/j.chstcc.2025.100220)
Supplement: 2 [file NIHMS2157069-supplement-2.docx]

**Follow Up Interview: Understanding Perspectives of Surrogate Decision Makers of Patients who Received Tracheostomies for Prolonged Mechanical Ventilation**

**Question guide:**

1. How are you doing?
2. How have things been going over the last X weeks/months?
   1. What has been going well?
   2. What has been challenging?
      1. How did you work through that?
3. After all that has happened, do you have any reflections about the decision to proceed with tracheostomy?
   1. What else would have been helpful to you at that time?
   2. What information do you wish you had known prior to tracheostomy? (i.e., risks, implications, prognosis, outcomes)
   3. What resources, services and/or support do you wish you had? When would these resources/services/support be helpful?
   4. Is there anything else you wish the health care team would have done differently? Anything you would have done differently?
4. Those are all the questions I have, is there anything else around this topic you think I should know about?

**Conclusion:** We really appreciate all your thoughts and input. All your comments and private and confidential. They are important for making care better for people like you and your family member. Thank you again.
