# Supplementary material for: Surrogate Perspectives on the Communication and Support Processes That Enable Them as Active Decision-Makers Across Chronic Critical Illness
Source: CHEST Crit Care. Author manuscript; Available in PMC 2026 Apr 9. (PMC13060019; doi:10.1016/j.chstcc.2025.100220)
Supplement: 3 [file NIHMS2157069-supplement-3.docx]

**Validated Instruments Included in Participant Survey**

Brief Health Literacy Screen[^1^](https://sciwheel.com/work/citation?ids=17198859&pre=&suf=&sa=0&dbf=0) is a 4-item measure designed to assess health literacy (degree to which one can read, understand, exchange, and use health information and resources). Each item is scored 1 to 5 based on the respondent’s answer, yielding a total score between 4 and 40. Scores are interpreted as follows: 4-12 indicates limited health literacy, 13-26 indicates marginal health literacy, and 17-20 indicates adequate health literacy.

Decision Regret Scale[^2^](https://sciwheel.com/work/citation?ids=1821118&pre=&suf=&sa=0&dbf=0) is a 5-item measure designed to assess distress or remorse after a healthcare decision. Items are rated on 5-point Likert scales. Total scores are transformed to a 0-100 scale for easier interpretation, with 0 indicating no regret and 100 indicating high regret.

Subjective Numeracy Scale[^3^](https://sciwheel.com/work/citation?ids=6596521&pre=&suf=&sa=0&dbf=0)^,^[^4^](https://sciwheel.com/work/citation?ids=6596525&pre=&suf=&sa=0&dbf=0) is an 8-item measure designed to assess individuals’ self-perceived ability to understand and work with numerical information, and their preferred presentation of numerical information. Items are rated on 6-point Likert scales. Total scores range from 8 to 48, with higher scores indicating higher subjective numeracy.

Wake Forest Physician Trust Scale[^5^](https://sciwheel.com/work/citation?ids=14939876&pre=&suf=&sa=0&dbf=0) is a 10-item measure designed to assess an individuals’ trust in their physicians. Items are rated on 5-point Likert scales. Total scores range from 5-50, with higher scores indicating more trust.

References:

[1.    Sand-Jecklin K, Coyle S. Efficiently assessing patient health literacy: the BHLS instrument. Clin Nurs Res. 2014 Dec;23(6):581–600. PMID: 23729022](https://sciwheel.com/work/bibliography/17198859)

[2.    Brehaut JC, O’Connor AM, Wood TJ, Hack TF, Siminoff L, Gordon E, Feldman-Stewart D. Validation of a decision regret scale. Med Decis Making. 2003;23(4):281–292. PMID: 12926578](https://sciwheel.com/work/bibliography/1821118)

[3.    Fagerlin A, Zikmund-Fisher BJ, Ubel PA, Jankovic A, Derry HA, Smith DM. Measuring numeracy without a math test: development of the Subjective Numeracy Scale. Med Decis Making. 2007 Oct;27(5):672–680. PMID: 17641137](https://sciwheel.com/work/bibliography/6596521)

[4.    Zikmund-Fisher BJ, Smith DM, Ubel PA, Fagerlin A. Validation of the Subjective Numeracy Scale: effects of low numeracy on comprehension of risk communications and utility elicitations. Med Decis Making. 2007 Oct;27(5):663–671. PMID: 17652180](https://sciwheel.com/work/bibliography/6596525)

[5.    Katz E, Edelstein B. Psychometric properties of the wake forest physician trust scale with older adults. Innov Aging. 2018 Nov 1;2(suppl_1):978–978. PMCID: PMC6239829](https://sciwheel.com/work/bibliography/14939876)
